# 

**Published:** 1850-11

**Authors:** 


### NOTICE TO CORRESPONDENTS.

Communications and Books for notice should be addressed to the Editor, care of Messrs. Lindsay & Blakiston.

Letters, &c., connected with the *business affairs* of the Journal should be addressed to the Publishers.

Papers for publication must be received *before* the 20th of the month, or they cannot appear in the forthcoming number.

The following Journals have been received in exchange:

New York Medical Gazette. Weekly.

The Boston Medical and Surgical Journal. (Weekly, Boston.)

Buffalo Journal.

Medical News.

American Journal of Insanity.

Southern Medical and Surgical Journal. Oct.

The British American Journal of Medical and Physical Science. (Montreal.)

The London Lancet. (Weekly, London.)

The Medical Times. Weekly, London.

Dublin Medical Press.

---

The following works have also been received for notice:

Copland on Apoplexy and Palsy. From Lea & Blanchard.

Howard on the Eye.

Spectacles, their Uses and Abuses. By Sichel.

Frick on Renal Diseases. From Lea & Blanchard.

An Essay on Zoo-Adynamia. By J. G. Ziegler, M. D., Philadelphia.

Fownes' Chemistry for Students. 3d edition. From Lea & Blanchard.

---

Communications have been received from:—

W. J. Reese, M. D., Alabama.

William Waters, M. D., Fredericktown, Md.

G. H. H. Koontz, Woodstock, Va.

E. C. Banks, Lawrenceville, Ill.

S. S. Hornor, Philadelphia.

J. M. Steiner, M. D., U. S. Army.

J. Travis, M. D., Tennessee.

---

The foreign correspondents of the Examiner will please direct their Exchanges and other communications to the care of Mr. Charles J. Skeet, 27 King William St., Charing Cross, London, or Mr. H. Bossange, 21 Bis, Quai Voltaire, Paris.

